# Intravitreal OZURDEX vs. Intravitreal Bevacizumab for Diabetic Macular Edema: A Comprehensive Review

**DOI:** 10.7759/cureus.56796

**Published:** 2024-03-23

**Authors:** Raina Jain, Sachin Daigavane

**Affiliations:** 1 Ophthalmology, Jawaharlal Nehru Medical College, Datta Meghe Institute of Higher Education and Research, Wardha, IND

**Keywords:** safety, efficacy, intravitreal, bevacizumab, ozurdex, diabetic macular edema

## Abstract

This comprehensive review examines the efficacy, safety, and implications of intravitreal OZURDEX and intravitreal bevacizumab in treating diabetic macular edema (DME). DME is a common complication of diabetes mellitus and a leading cause of vision loss. OZURDEX, through sustained release of dexamethasone, targets inflammation and vascular permeability, while bevacizumab inhibits vascular endothelial growth factor (VEGF), reducing angiogenesis. However, differences in safety profiles exist, with OZURDEX associated with an increased risk of intraocular pressure elevation and cataract formation and bevacizumab potentially carrying systemic risks. The choice between these treatments should be individualized, considering patient preferences, ocular and systemic comorbidities, and cost-effectiveness. Collaboration among healthcare providers is essential for the comprehensive management of DME. Future research should focus on long-term comparative studies, predictors of treatment response, and exploration of novel therapeutic targets to optimize treatment outcomes for patients with DME.

## Introduction and background

Diabetic macular edema (DME) is a serious complication of diabetic retinopathy, a condition affecting the blood vessels of the retina in individuals with diabetes. DME involves explicit fluid accumulation in the macula, the central part of the retina responsible for sharp, central vision. This fluid buildup can lead to blurred or distorted vision and, if left untreated, may progress to severe vision loss or blindness [1]. The treatment of DME is of paramount importance due to its potential to cause significant vision impairment and adversely affect the quality of life for individuals living with diabetes. Prompt and effective management can help preserve vision, prevent further deterioration, and enhance overall visual function, thus enabling patients to maintain independence and engage in daily activities [2].

Intravitreal injections are a cornerstone of modern ophthalmic therapy, particularly in treating retinal diseases such as DME. These injections involve direct medication delivery into the eye's vitreous cavity, bypassing systemic circulation and providing concentrated therapy to the target tissues within the retina [3]. Intravitreal injections offer several advantages, including high drug bioavailability, precise dosing, and reduced systemic side effects compared to oral or systemic administration [4].

OZURDEX (dexamethasone (DEX) intravitreal implant) and bevacizumab (Avastin) are widely utilized intravitreal agents for treating DME. OZURDEX is a sustained-release biodegradable implant containing DEX, a potent corticosteroid with anti-inflammatory and anti-edema properties [5]. The implant gradually releases DEX over several months, providing sustained therapeutic effects. Bevacizumab is a monoclonal antibody that targets vascular endothelial growth factor (VEGF), a key mediator of vascular leakage and angiogenesis in DME. By inhibiting VEGF activity, bevacizumab helps reduce macular edema and improve visual outcomes in affected individuals [6]. This review compares the efficacy, safety, and outcomes of intravitreal OZURDEX vs. intravitreal bevacizumab for treating DME. By synthesizing existing evidence and clinical data, this review seeks to provide insights into these treatment modalities' relative merits and limitations, aiding clinicians in informed decision-making and optimizing patient care.

## Review

Pathophysiology of diabetic macular edema

Microvascular Changes in Diabetes

Microvascular changes in diabetes encompass a spectrum of complications affecting diverse organs and tissues. Both type 1 and type 2 diabetes can exert detrimental effects on the microvasculature across multiple organs, precipitating tissue dysfunction and organ impairment [7,8]. While retinopathy, nephropathy, and neuropathy are the classic microvascular pathologies linked with diabetes, other tissues, such as the brain, myocardium, and skin, may also be impacted [7,9]. Hyperglycemia is pivotal in instigating vascular complications in diabetes through various metabolic pathways, including the generation of advanced glycation end products, aberrant activation of signaling cascades, heightened production of reactive oxygen species, and dysregulated stimulation of hemodynamic regulatory systems [8,9].

The pathophysiology underlying microvascular complications in diabetes entails prolonged exposure of tissues to hyperglycemia, culminating in oxidative stress, formation of advanced glycation end products, and subsequent tissue damage [9]. Diabetic retinopathy stands as a prevalent microvascular complication capable of causing vision loss and blindness owing to microvascular damage within the retina induced by hyperglycemia [9,10]. Timely detection and management of microvascular complications are imperative in impeding further progression and mitigating associated risks. Rigorous glycemic control, proactive blood pressure regulation, lipid-lowering interventions, and regular screening for complications emerge as indispensable strategies for preventing and addressing microvascular complications in diabetes [9,10].

Inflammatory Mediators

In the pathogenesis of DME, inflammatory mediators play a pivotal role. Prolonged hyperglycemia triggers the disruption of the blood-retinal barrier (BRB) by fostering the accumulation of advanced glycated end products, thereby causing interstitial fluid buildup within and beneath the retina [11]. Consequently, this disruption prompts the leakage of various molecules, including VEGF, interleukins (ILs), matrix metalloproteinases (MMPs), and tumor necrosis factor (TNF), which induce inflammation, oxidative stress, and vascular dysfunction [11]. Notably, the research underscores the inflammatory component inherent in DME, with an array of chemokines and cytokines implicated in its pathogenesis [11]. Among these factors, VEGF, ILs, MMPs, and TNF are prominent, upregulating multiple pathways that exacerbate inflammation and vascular dysfunction [11]. Additionally, alterations in the neurovascular unit and changes in retinal vascular permeability further contribute to the development of DME [11]. A comprehensive understanding of the involvement of inflammatory mediators in DME is imperative for developing targeted therapies capable of addressing specific steps within the inflammatory cascade, thereby improving patient outcomes and preserving vision. Presently, ongoing research endeavors are exploring novel drug targets associated with these mediators to broaden the therapeutic armamentarium available for patients with DME [12].

Vascular Endothelial Growth Factor Pathway

The VEGF pathway is pivotal in angiogenesis and vascular permeability regulation. VEGF binds to VEGF receptors on endothelial cells, particularly VEGF receptor 2 (VEGFR-2) (fetal liver kinase-1/kinase insert domain-containing receptor (Flk-1/KDR)), initiating a tyrosine kinase pathway that culminates in angiogenesis [13]. Acting as a signaling protein, VEGF stimulates the formation of blood vessels and contributes to various physiological processes such as wound healing, hematopoiesis, and tissue development [13]. Within the VEGF family, growth factors play essential roles in vasculogenesis and restoring oxygen supply to tissues under conditions of inadequate blood circulation [13]. The signaling cascade of VEGF involves the activation of numerous downstream pathways after the binding of VEGF ligands to their receptors, thereby eliciting gene expression induction, regulation of vascular permeability, cell migration, proliferation, and survival [14]. This pathway encompasses the activation of phospholipase Cγ (PLCγ)-protein kinase C (PKC)-mitogen-activated protein kinase (MAPK) for angiogenesis and regulation of vascular permeability [15]. In pathological contexts, elevated levels of VEGF can instigate the formation of pathological vessels through angiogenesis, impacting conditions such as ischemic stroke, central nervous system (CNS) injury, and neurodegenerative diseases like Alzheimer's disease [16].

Mechanisms Underlying Edema Formation

The mechanisms underlying edema formation, particularly in macular edema, involve a multifaceted interplay of factors. In macular edema, fluid accumulation within the central retina stems from various pathological processes that affect the integrity of the BRB and culminate in heightened osmotic pressure and subsequent fluid buildup [17-19]. Dysfunction of the BRB permits the infiltration of proteins and other solutes into the retinal tissue, disrupting the delicate balance and resulting in edema [17-19]. Numerous pivotal factors contribute to the onset of macular edema, encompassing vascular components, compromised BRB function, elevated osmotic pressure, and the involvement of inflammatory mediators such as VEGF and prostaglandins [17-19]. The accumulation of fluid in the macula can precipitate vision loss and irreversible visual impairment if left untreated [18]. Various patterns of macular edema have been delineated, including cystoid macular edema, diffuse macular edema, and subretinal detachment, each exerting its influence on retinal architecture and visual acuity [18].

Intravitreal OZURDEX

Description and Mechanism of Action

OZURDEX® is a sustained-release, biodegradable steroid implant comprising DEX, a potent corticosteroid. DEX mitigates inflammation by suppressing multiple inflammatory cytokines, resulting in reduced edema, fibrin deposition, capillary leakage, and the migration of inflammatory cells. This mechanism of action renders OZURDEX® advantageous in the treatment of various conditions, including DME, macular edema following retinal vein occlusion (RVO), and non-infectious posterior segment uveitis [20]. The NOVADUR® technology utilized in OZURDEX® employs a proprietary drug delivery system characterized by a solid polymer matrix containing 0.7 mg of DEX. Over time, this matrix biodegrades into lactic acid and glycolic acid. Administration of OZURDEX® involves an injection performed in-office using a patented intravitreal applicator. These sterile, single-use applicators come preloaded with the implant and incorporate a second-generation needle to facilitate precise administration via a shelved injection technique [21].

Pharmacokinetics

The pharmacokinetics of intravitreal OZURDEX, a sustained-release DEX implant, have been extensively investigated. In a study by Chang-Lin et al., the release profile of DEX in the retina and vitreous humor was monitored for up to six months following administration in monkeys. DEX was detected in these tissues throughout the six months, with peak concentrations observed during the initial two months. Additionally, the study revealed a threefold upregulation in cytochrome P450 3A8 (CYP3A8) gene expression in the retina up to six months post-implantation [22]. Another study by Chang-Lin et al. examined the pharmacokinetics of DEX after OZURDEX implantation in nonvitrectomized and vitrectomized eyes of rabbits. The findings indicated the presence of DEX in both types of eyes for a minimum of 31 days, with no notable disparities in DEX concentration between nonvitrectomized and vitrectomized eyes. Moreover, the vitreoretinal pharmacokinetic profiles exhibited similarity between the two groups, thereby corroborating with clinical observations of OZURDEX in patients who have undergone vitrectomy [23].

Clinical Trials and Efficacy

Intravitreal OZURDEX has effectively treated DME across various clinical trials. A three-year randomized, sham-controlled trial revealed sustained clinically significant improvements in vision with OZURDEX throughout the study duration [24]. In a pooled analysis of all phakic DME randomized patients from two multicenter, masked, randomized, sham-controlled studies, the primary endpoint was the proportion of patients achieving 15 or more letters' improvement in best-corrected visual acuity (BCVA) from baseline at month 39 [24]. OZURDEX was also effective in pseudophakic patients, leading to a significant improvement in BCVA from baseline. However, cataracts in phakic patients negatively impacted visual acuity during the study [24]. In a systematic review and meta-analysis comparing the efficacy of intravitreal DEX implant (OZURDEX) vs. anti-VEGF therapies, no significant differences in BCVA change were observed between OZURDEX and anti-VEGF therapies in patients with non-resistant DME [25]. However, when considering baseline central retinal thickness (CRT), the meta-regression analysis indicated that a baseline CRT exceeding 410 µm could be a critical factor influencing the anatomical outcome in favor of OZURDEX therapy [25]. Regarding safety, pooled results from two multicenter, masked, randomized, sham-controlled studies revealed no significant difference in intraocular pressure (IOP) change between the OZURDEX and anti-VEGF groups [24]. Nonetheless, cataracts in phakic patients could impact visual acuity during the study [24].

Safety Profile

The safety profile of intravitreal OZURDEX requires critical consideration from patients. It should be avoided in eye infections, advanced glaucoma, torn posterior lens capsules, or allergies to any components. Injections, such as OZURDEX, are associated with potential risks of severe eye infections, inflammation, increased eye pressure, and retinal detachments, necessitating regular monitoring by an eye doctor [26]. Corticosteroids like OZURDEX may also contribute to cataracts, increased eye pressure, glaucoma, and secondary eye infections. Patients with a history of ocular herpes simplex should inform their doctor, as corticosteroids are not recommended in such cases [27]. During the injection procedure of OZURDEX, patients may experience pressure and hear a click when the implant is released into the eye. Temporary visual blurring may occur post-injection, cautioning patients to refrain from driving until vision resolves. Repeated treatments with OZURDEX may predispose to cataract formation and increased eye pressure, necessitating ongoing monitoring by a healthcare provider [28]. Studies have demonstrated the effectiveness of OZURDEX in enhancing visual acuity and reducing CRT in patients with macular edema related to RVO. While adverse events, such as elevated IOP, cataract progression, and cataract formation, have been reported, they are generally manageable [29]. The choice between OZURDEX and anti-VEGF treatments depends on factors such as efficacy in reducing macular edema and safety profiles concerning adverse events like cataract formation and elevated IOP [29].

Cost Considerations

The cost of intravitreal OZURDEX varies considerably depending on the country and the specific healthcare system. In the United States, for instance, a single 0.7 mg DEX implant costs approximately $1,455 [30]. Contrarily, in Spain, treating patients with naïve DME with DEX instead of aflibercept is reported to be €77,349 more expensive [31]. From the Andalusian Regional Healthcare Service (ARHS) perspective, the total cost of one year of OZURDEX treatment in treatment-naïve eyes was significantly lower than in previously treated eyes [32]. The cost-effectiveness of OZURDEX in the treatment of DME has been subject to evaluation in various studies. According to cost-effectiveness analysis, DEX implants had a 41% probability of being cost-effective at a willingness-to-pay threshold of $50,000, with the sponsor's submitted price for OZURDEX being $1,446.03 per 0.7 mg DEX implant [33].

Intravitreal bevacizumab

Description and Mechanism of Action

Bevacizumab, marketed under Avastin, is a monoclonal antibody renowned for inhibiting angiogenesis by binding and neutralizing VEGF (VEGF-A). This mechanism of action disrupts VEGF's interaction with its cell surface receptors, thereby impeding the process of angiogenesis. Bevacizumab received FDA approval in 2004 as the pioneering antiangiogenic agent for specific cancer types [34]. In ophthalmology, intravitreal bevacizumab is employed off-label to address conditions, such as diabetic macular and macular edema, stemming from RVOs. Research substantiates its efficacy in enhancing visual acuity and mitigating macular edema by targeting VEGF-mediated pathways [35]. The safety profile of bevacizumab encompasses rare ocular and systemic adverse effects, including transient elevation of IOP, subconjunctival hemorrhage, uveitis, acute rise in blood pressure, and mild allergic reactions. Despite these potential side effects, bevacizumab remains a valuable treatment option in oncology and ophthalmology due to its profound capability to inhibit angiogenesis by targeting VEGF [34].

Pharmacokinetics

The pharmacokinetics of intravitreal bevacizumab have been extensively investigated, particularly in animal models such as rabbits. Studies have revealed that following intravitreal injection of 1.25 mg/0.05 mL of bevacizumab, the maximum concentration in the vitreous and aqueous humor is typically attained within the first day post-injection, with concentrations remaining elevated for up to 29 days afterward [36,37]. In rabbits, the vitreous half-life of 1.25 mg intravitreal bevacizumab is approximately 4.32 days, and concentrations of >10 µg/mL are maintained in the vitreous humor for up to 30 days [36]. Moreover, research indicates that humans possess a larger vitreous cavity than rabbits, which may influence the pharmacokinetics of bevacizumab in human eyes. Human studies have demonstrated that following a single intravitreal injection of 1.5 mg bevacizumab, the drug concentration in the aqueous humor peaks on the first-day post-injection and declines over time [36]. Additionally, studies have highlighted the efficient diffusion of bevacizumab through retinal layers into the subretinal space, underscoring its utility in treating conditions such as macular edema [36].

Clinical Trials and Efficacy

Several studies have investigated intravitreal bevacizumab's efficacy for treating retinopathy of prematurity (ROP). One study on stage 3+ ROP demonstrated increased efficacy compared to conventional laser therapy [38]. Another trial concentrated on threshold ROP and found that intravitreal bevacizumab effectively treated stage 2+ or stage 3+ ROP in zone I or zone II, with the primary objective being the regression of ROP [39]. In a randomized clinical trial comparing intravitreal therapy with ranibizumab, aflibercept, and bevacizumab for macular edema secondary to central RVO, aflibercept was deemed non-inferior to ranibizumab. However, the noninferiority of bevacizumab regarding vision outcomes remained inconclusive [40]. Furthermore, a phase II randomized clinical trial evaluated the short-term effects of intravitreal bevacizumab for DME. The findings revealed that approximately half of the eyes exhibited an initial positive response to bevacizumab. However, further long-term studies are warranted to establish definitive conclusions regarding the safety and effectiveness of this treatment approach [41].

Safety Profile

The safety profile of intravitreal bevacizumab (Avastin) encompasses numerous warnings and precautions concerning potential adverse effects. These may include gastrointestinal perforation, complications related to surgery and wound healing, hemorrhage, arterial and venous thromboembolic events, hypertension, posterior reversible encephalopathy syndrome, renal injury, proteinuria, infusion-related reactions, embryo-fetal toxicity, ovarian failure, fertility issues, congestive heart failure, and more [42]. Studies have generally indicated that intravitreal bevacizumab is safe and well-tolerated in the treatment of various ocular disorders, with rare reports of systemic adverse events [43]. The incidence of severe ocular adverse effects associated with bevacizumab is low, and transient elevation of IOP is a common occurrence that typically resolves rapidly [44]. In a study examining the safety profile of intravitreal bevacizumab in patients with diverse eye conditions, it was observed that rates of serious adverse events were low compared to other intravitreal treatments, sham injections, and laser therapy. However, existing studies faced limitations such as small sample sizes and unclear reporting of safety outcomes [45]. Real-world evidence also corroborates the safety and cost-effectiveness of intravitreal bevacizumab as a pharmacotherapeutic agent for ocular disorders [43].

Cost Considerations

Cost considerations are pivotal in determining the choice of treatments, including intravitreal bevacizumab, for eye conditions. Studies have conducted comparisons of the cost-effectiveness of bevacizumab with other treatments like ranibizumab, revealing that continuous use of bevacizumab may cost approximately £30,220 per quality-adjusted life year (QALY) gained compared to discontinuous use [46]. The cost of administering intravitreal injections of bevacizumab or ranibizumab is estimated at around £61 per injection, with additional expenses for monitoring consultations and fluorescein angiography [46]. Bevacizumab emerges as a more cost-effective option than ranibizumab, resulting in notable cost savings, as demonstrated in various studies [47]. Moreover, implementing bevacizumab vial sharing has yielded significant cost reductions in treating retinal diseases, contributing to substantial savings in healthcare expenses [48]. Ophthalmologists encounter challenges in managing the financial aspects associated with the use of expensive drugs like ranibizumab and aflibercept, which can amount to approximately $1,850 to $2,023 per dose, in contrast to the more economically feasible bevacizumab priced at approximately $50-$60 per dose [47]. Patients' decisions regarding treatment options are influenced by factors such as cost and effectiveness, with bevacizumab often being preferred for its cost-effectiveness despite exhibiting similar clinical outcomes to other anti-VEGF therapies [49].

Clinical guidelines and recommendations

Current Guidelines for the Treatment of DME

Current guidelines for the treatment of DME advocate for intravitreal anti-VEGF therapy as the primary treatment strategy. In cases where patients do not exhibit adequate responses to anti-VEGF therapy, switching to intravitreal corticosteroid therapy, such as OZURDEX, is recommended. According to the EURETINA guidelines, topical corticosteroid therapy is suggested for nonresponders who have undergone three to six anti-VEGF injections [50]. Furthermore, it is recommended to evaluate the response to intravitreal corticosteroid treatment at an eight-week interval based on findings from phase III studies involving DEX and fluocinolone acetonide implants, with a consideration to switch to corticosteroid therapy as necessary [51]. OZURDEX is indicated as an alternative treatment option for DME patients who exhibit suboptimal responses to anti-VEGF therapy [52]. Proper preparation, including anesthesia and the use of a broad-spectrum microbicide, is stressed before administering intravitreal therapy to ensure safety during injections [25].

Recommendations Regarding the Use of OZURDEX and Bevacizumab

The utilization of OZURDEX and bevacizumab for managing DME is guided by clinical evidence and recommendations. Studies comparing the pain associated with intravitreal OZURDEX injections vs. bevacizumab injections revealed no significant difference in pain levels between the two treatments. Despite OZURDEX injections utilizing a larger needle gauge and tunneled injection technique, they were not linked to increased pain compared to bevacizumab injections, potentially enhancing patient compliance with long-term treatment regimens [53]. Clinical guidelines suggest that while intravitreal anti-VEGF therapy is advocated as the first-line treatment for DME, OZURDEX can be considered an option for patients with suboptimal responses to anti-VEGF therapy. It is recommended to evaluate the response to corticosteroid treatment at an eight-week interval and contemplate switching to corticosteroid therapy when necessary, based on phase III studies involving DEX and fluocinolone acetonide implants [53]. Additionally, the National Institute for Health and Clinical Excellence (NICE) issued recommendations endorsing the use of OZURDEX for DME patients whose response to anti-VEGF therapy is suboptimal, underscoring its role as an alternative treatment approach in clinical practice [54].

Future directions and emerging therapies

Potential Advancements in DME Treatment

Vabysmo (faricimab): Vabysmo, which received approval in February 2022, represents a groundbreaking advancement in treating DME. Unlike traditional therapies targeting a single disease pathway, Vabysmo uniquely targets two pathways contributing to DME. This innovative approach offers a significant advantage in managing the condition effectively. Additionally, Vabysmo offers the convenience of injections every four weeks initially, potentially extending treatment intervals up to 16 weeks. This extended dosing schedule provides patients with a more convenient and manageable treatment regimen, potentially enhancing treatment adherence and improving overall outcomes [55].

Individualized management: The future of DME management is shifting toward personalized approaches tailored to meet each patient's specific needs. This individualized management strategy recognizes the heterogeneity of DME and aims to optimize treatment outcomes by considering disease severity, response to therapy, comorbidities, and patient preferences. By customizing treatment plans based on individual characteristics, healthcare providers can maximize therapeutic efficacy and minimize potential adverse effects, improving patient outcomes [56].

Novel agents: Ongoing research explores many novel agents for treating DME. These include new anti-VEGF drugs, IL inhibitors, rho-kinase inhibitors, and neuroprotective agents. These emerging therapies offer promising avenues for more effective management of DME by targeting various pathways involved in the pathogenesis of the disease. By expanding the therapeutic armamentarium, clinicians can better address the diverse needs of patients with DME and potentially improve treatment outcomes [56].

**Figure 1:**
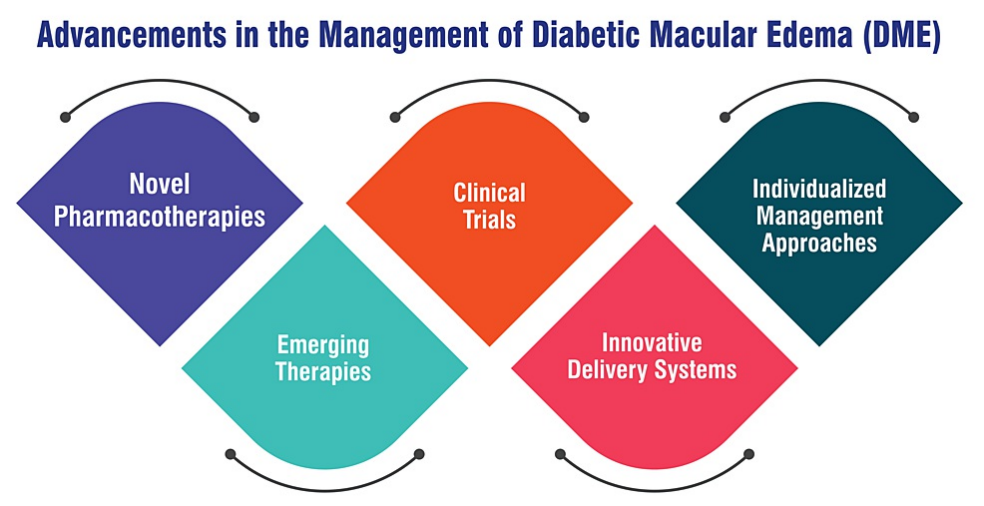
Advancements in the management of DME This figure is self-created by the corresponding author.

Innovative delivery systems: Current investigations focus on innovative delivery systems to enhance the efficacy and convenience of DME treatment. These include gene therapies and implantable devices designed for sustained drug release. By harnessing advanced delivery technologies, such as sustained-release implants and targeted gene therapies, researchers aim to revolutionize DME treatment by offering more convenient, efficient, and patient-friendly therapeutic options. These advancements can potentially transform the landscape of DME management and improve patient outcomes [55]. Advancements in the management of DME are shown in Figure 1.

Investigational Therapies and Ongoing Research

New treatment modalities: Ongoing clinical trials represent a dynamic frontier in managing diabetic retinopathy and DME. These trials explore novel pharmacotherapies and treatment protocols aimed at introducing innovative modes of treatment, genetic applications, and advancements in anti-VEGF therapy. By pushing the boundaries of traditional treatment approaches, researchers aspire to develop more effective and durable treatment options for patients with diabetic retinopathy and DME. These efforts aim to enhance treatment outcomes and improve the long-term prognosis for affected individuals [57,58].

**Figure 2:**
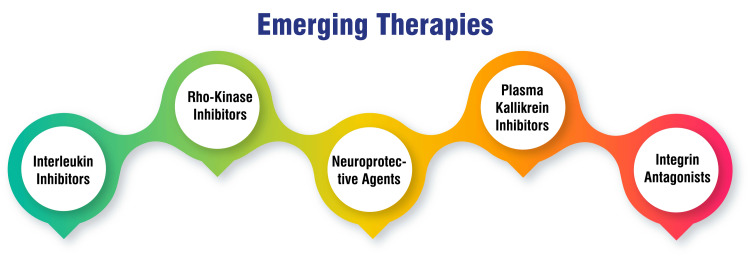
Emerging therapies of DME This figure is self-created by the corresponding author.

Emerging therapies: Research efforts are intensifying toward developing investigational agents targeting new pathways involved in the pathogenesis of DME. These include IL, rho-kinase inhibitors, neuroprotective agents, plasma kallikrein, and integrin antagonists. By exploring these emerging therapies, researchers aim to broaden the therapeutic armamentarium for DME and improve treatment efficacy for patients who may not respond optimally to existing therapies. These investigational agents hold promise in addressing the diverse underlying mechanisms contributing to DME, thus offering new avenues for therapeutic intervention and enhanced patient outcomes [59,60]. Emerging therapies are shown in Figure 2.

Clinical trials: EyePoint Pharmaceuticals has embarked on the Phase II VERONA clinical trial evaluating the potential of EYP-1901 as a treatment for DME. This investigational therapy utilizes vorolanib for sustained delivery and aims to assess its efficacy in patients who have previously undergone standard anti-VEGF therapy. EYP-1901 can transform the treatment landscape for VEGF-mediated retinal diseases, such as DME, by offering a novel therapeutic approach. Through rigorous clinical evaluation, researchers seek to validate the efficacy and safety of EYP-1901, paving the way for its potential incorporation into clinical practice and improving treatment outcomes for patients with DME [61].

Future directions: The latest research endeavors also explore innovative delivery systems to optimize the delivery of therapeutic agents for DME. These include gene therapies and implantable devices designed for sustained drug release. By leveraging these advanced delivery systems, researchers aim to revolutionize DME treatment by offering more convenient and effective therapeutic options. Additionally, individualized management approaches tailored to each patient's specific needs are emphasized. By considering factors, such as disease severity, treatment response, and patient preferences, personalized treatment plans can be developed to optimize treatment outcomes and improve the overall quality of care for individuals with DME [59].

## Conclusions

In conclusion, comparing intravitreal OZURDEX and intravitreal bevacizumab for treating DME reveals critical insights for clinical practice and future research directions. Both treatments have demonstrated efficacy in improving visual acuity and reducing macular edema, with OZURDEX targeting inflammation and vascular permeability through sustained release of DEX. At the same time, bevacizumab inhibits VEGF, thereby reducing angiogenesis. However, their safety profiles differ, with OZURDEX associated with an increased risk of IOP elevation and cataract formation and bevacizumab potentially carrying systemic risks. Clinicians should carefully consider individual patient characteristics and preferences when selecting between these treatments, emphasizing the importance of shared decision-making and patient education. Long-term comparative studies, exploration of predictors of treatment response, and investigation into novel therapeutic targets and delivery systems are recommended for advancing the management of DME and improving patient outcomes. Collaborative efforts among healthcare providers are crucial for comprehensive care and optimal treatment outcomes for patients with DME.
